# Whitening toothpastes effect on nanoparticle resin composite roughness after a brushing challenge: An *in vitro* study

**DOI:** 10.4317/jced.55533

**Published:** 2019-04-01

**Authors:** José-Handerson-Araújo dos Santos, Natyla-Maysa-de Lima Silva, Mário-Gilson-Nina Gomes, Marco-Aurélio-Benini Paschoal, Isabella-Azevedo Gomes

**Affiliations:** 1 Undergraduate Student of the Dentistry, Universidade CEUMA, São Luís, MA, Brazil; 2 Professor of Dentistry, Universidade CEUMA, São Luís, MA, Brazil; 3 Department of Pediatric Dentistry and Orthodontics, Federal University of Minas Gerais - UFMG, Belo Horizonte - MG, Brazil

## Abstract

**Background:**

Nowadays, the use of whitening toothpastes is a common habit, especially among young adults, due to aesthetic appeal. On the other hand, little is known regarding the effects of brushing with those newly dentifrices on wear properties of resin composites.

**Material and Methods:**

Thirty specimens of nanoparticle composite resin were fabricated and stored in distilled water for 24 h at 370C. After this, the roughness analysis was performed and submitted to the simulated brushing technique using three types of toothpastes: conventional (GI), and two with whitening effect (GII and GIII) for a period of 15 days, with 2 brushing sessions per day for 2 minutes each. The final surface roughness was analyzed after completing all the brushing cycles and stereoscopic images were taken for each group. The data was analyzed by one-way ANOVA and Tukey-test post hoc for intergroup comparison and the T-test for dependent samples as well (α = 0.05).

**Results:**

However showing an increase of roughness for all groups after the brushing cycles (*p* = 0.01), no statistically significant differences among the groups after simulated brushing was verified (*p* = 0.17). Yet, just some cracks of the stereoscopic images were shown, demonstrating no distinct visual effects among the studied groups.

**Conclusions:**

After simulated brushing with the whitening toothpastes, similar degree of roughness was verified on the composite resin tested.

** Key words:**Composite resin, toothpastes, whitening.

## Introduction

Esthetic rehabilitation in dentistry has become a demand in the globalized world. Presenting white, well-shaped, well cared for and well aligned teeth means that not only have the esthetic demands been met - since these conditions are important indicators of oral health - but that the requirements of the contemporary world and its high levels of sociability have also been met (1).

People, in general, desire to have a harmonious smile, and dental whitening has become essential among the basic standards of esthetic, according to the perspective of ethnocentrism. The ideology of whitening presents white as a model of beauty and success. Moreover, the advances of technology and research in the field of cosmetics at present has allowed refinement and perpetuation of dental whitening (2,3).

The indiscriminate use of whitening agents may cause morphological changes that compromise the superficial integrity of enamel (4,5), increase in roughness, changes in inorganic composition (6,7), reduction in microhardness (8) and in the mineral content of enamel (9).

More recently, different products have been launched on the marked, among them toothpastes and mouth washes, which are easily found in drug stores and supermarkets with main purpose to provide a practical, fast, easy and low-cost whitening effect. They are designed as over-the-counter (OTC) products that require no supervision or indication by a dental professional. In the toothpastes, the concentration of peroxide is relatively low, at times nonexistent, to the point of allowing the whitening power they have to be questioned (10,11); aided by the presence of abrasives, among them, calcium carbonate and silica, although others may be present that could damage the enamel structure (12,13).

When whitening is performed on a restored tooth, changes may occur in the restorative material and enamel, however, the restorations may not necessarily have to be replaced after whitening. Analysis of the surface and interface between the restorative material and enamel after dental whitening is fundamental, considering that the quality of this relationship is of great relevance to the longevity of restorations (14).

At present, the use of these whitening agents is so widespread that many individuals use them randomly, without being monitored by a dentist. Therefore, there are a growing number of researches to confirm whether whitening toothpastes may cause undesirable effects on soft, hard, and restored tissues as well (15).

Surface roughness is the set of microgeometrical irregularities that result in a surface, which arise from the interaction with processes of wear and are formed of numerous grooves and scratches that are more or less variable in shape, direction and depth (16).

Taking into consideration the growing number of persons that make use of these products, the aim of this study was to investigate whitening toothpastes and their effect on the surface roughness of a nanoparticle resin composite after a brushing challenge.

## Material and Methods

-Sample

After a pilot study, the sample size calculation consisted of a chance of 80% to detect a 25% of change difference after abrasive cycling between the toothpastes groups at 5% level of significance, requiring 10 samples of each group to perform this investigation. Thus, the study consisted a total of 30 test specimens made of nanoparticle resin composite (Filtek Z350, 3M ESPE, Sumaré, SP, Brazil) shade A2, divided into 3 Groups (N = 10), according to studied toothpastes types ( Table 1).

**Table 1:**
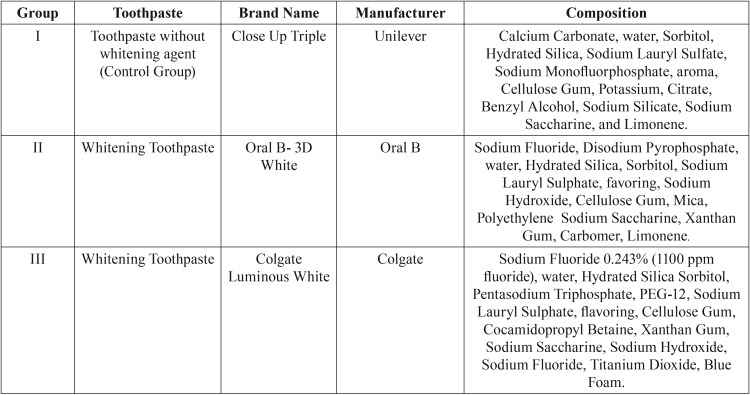
List of toothpastes and its composition.

-Specimen Preparation

Initially, the resin test specimens Filtek Z350 (3M) (Sumaré, São Paulo, Brazil), Shade A2, were fabricated in a circular steel matrix, measuring 10 mm X 2 mm (diameter and thickness). The composite was inserted into the matrix on a glass plate in a single increment. A polyester strip was placed over the resin inserted in the matrix and pressed down by a glass slide (Labor Import, Osasco, SP, Brazil), to obtain a flat surface. Subsequently, light polymerization was performed on the polyester strip and glass slide for 40 seconds with a LED light polymerizing appliance of 1250 mW/cm2 (Schuster Emitter A FIT, Santa Maria, RS, Brazil). After fabrication, the test specimens were removed from the metal matrix, stored in distilled water and transferred to a bacteriological oven at 37 ± 1°C for 24h. After this period, the test specimens were submitted to the finishing and polishing technique (Soflex- Pop On, 3M ESPE, Sumaré, SP, Brazil). During the finishing/polishing procedures, medium grain abrasive discs were used with movements in a single direction and control of pressure, for 40 seconds, performed by a single operator. The change of discs after every 3 resins test specimens was standardized. On conclusion of the finishing and polishing technique, the specimens were randomly divided into three groups for surface roughness analysis.

-Roughness analysis

After specimen preparation, the superficial roughness (initial Ra) of the samples was analyzed by using a rugosimeter (Mitutoyo Corporation, Japan) and the value was expressed as the arithmetic roughness value (Ra = µm).

The readout value was obtained by means of the arithmetic mean of three consecutive readouts on each test specimen, with each sample being carefully dried with absorbent paper before taking the readouts. For readouts, the ISO Standard 1997 specifications were used, whereby the test specimens were submitted to readouts in a cooled room with controlled temperature and humidity. The cut-off value used was 0.8 at the speed of 0.5 mm/s (17).

After this, the specimens were submitted to the simulated brushing model, and at the final of the entire process, a new roughness analysis was performed, considered as the final measurement (Final Ra).

-Simulated Brushing Model

The test specimens of the three studied groups were submitted to simulated brushing in a Brushing Machine XY (BIOPDI, São Carlos, SP, Brazil) for 500 cycles for 2 minutes with the use of a toothpaste/distilled water mixture (Colgate Palmolive - Divisão Kolynos do Brasil Ltda., Osasco, Brazil), used in the ratio of 1:1. To this, brushes with soft bristles (Colgate Palmolive - Divisão Kolynos do Brasil Ltda., Osasco, Brazil) were adapted to perform this test. Simulated brushing was performed twice a day for 2 minutes during 15 consecutive days. Between brushing cycles, the test specimens were transferred to a distilled water solution and stored in a bacteriological oven at 37oC. After the last brushing cycle of the time, the final roughness readout was taken, as previously described.

-Stereoscopic microscopy images

The test specimens were submitted to qualitative analysis by means of images captured by a stereoscopic microscope (Stereo Microscope Kozo Optical and Electronic Instrumental, Najing, China) and analyzed at 40x magnification.

-Statistical analysis

The data were tested for normality by using the Shapiro Wilk test (*p* < 0.05). For intergroup comparison of the initial and final roughness values, the Analysis of Variance (ANOVA), followed by the Tukey-test were used. For evaluating the roughness before and after the abrasive test for each group, the T-test for dependent samples was applied. For both statistical tests, the level of significance of 5% was used. The software program SPSS for Windows, version 23.0 (IBM, Armonk, NY, USA) was used for sample size calculation and statistical analysis.

## Results

 Table 2 shows the initial and final roughness values and standard deviation of each group.

**Table 2:**
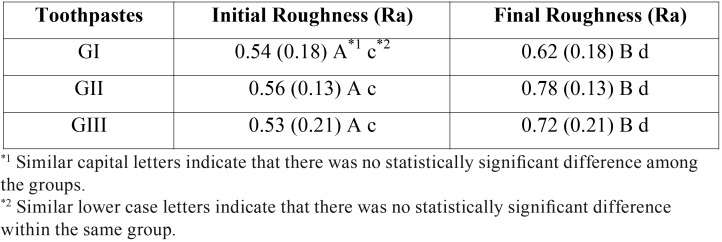
Values of roughness measurements of all groups before and after the simulated brushing challenge.

The results presented showed that no statistically significant differences were shown between the initial roughness values (*p* = 0.94), and also neither between the final roughness values (*p* = 0.17) of the different groups under study. With regard to the initial and final roughness values after the simulated brushing technique, statistically significant difference was found, with increase in roughness for all the studied groups (*p* = 0.01).

**Figure 1:**
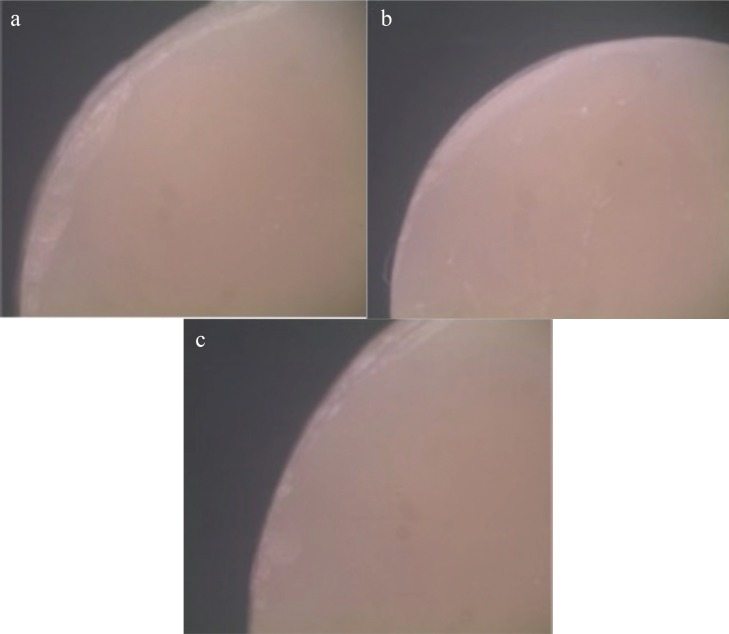
Stereoscopic images of all studied groups after 15 days of brushing cycling. A. Group I (control group – Close Up). B. Group II (Oral B- 3D White). C. Group III (Colgate Luminous White).

Relative to the images obtained by means of stereoscopic lens, after the abrasive cycle of simulated brushing, cracks were found at 40x magnification, which were observed for all the studied groups (Fig. 1).

## Discussion

The clinical wear of a resin composite restoration may result from innumerable factors, such as centric / functional contacts and friction due to food types and interproximal contacts. Among different kind of wear, abrasion by toothbrushing has been the most important issue that affects dental materials including resin composites (18).

In the present study, regardless of the toothpaste type, a statistical difference roughness values was verified after the brushing challenge ( Table 2). According to Quirynen *et al.* (19), brushing movements are able to compromise the finishing and polishing of composite restoration, causing wear and increasing the surface roughness composite. Still, a rougher surface interferes in the shine, material aesthetic (20) and facilitating bacterial plaque accumulation, supporting the development of secondary caries and gum diseases as well. Besides, Pinto *et al.* (6) observed that different toothpastes compositions presented a direct influence on the enamel and resin composite surface roughness, as stated by the reached results.

New toothpastes have been launched in the market with principal purpose of aesthetics improvement. The main whitening effect is based on interaction between peroxide compounds, surfactants, polyphosphates, enzymes in combination with abrasive substances (11,21,22). This investigation evaluated the effect of three toothpastes containing different abrasives: calcium carbonate (Close-Up Triple), hydrated silica (Oral B-3D White) and titanium dioxide (Colgate Luminous White) on a nanoparticle composite resin. Although different agents have been tested, no statistical differences regarding final roughness values neither the aspects of the performed images were verified (Figures A, B and C) for all evaluated toothpastes. Similar outcomes were obtained by some studies (23,24) using a dentifrice containing calcium carbonate proving to be the lesser abrasive than other whitening dentifrices. On this same way, silica and oxides compounds are categorized the most abrasive compounds in comparison to carbonate (25), as verified in this present investigation. Therefore, all dentifrices were able to increase the surface roughness, demonstrating that even using a less damaging compound, it could modify an important material property.

At analyzing the results, abrasiveness counterparts of dentifrices, such as hardness, size, number, distribution of the particles and ash content should be evaluated and considered as well. The cristaline form of tested abrasives demonstrated a diverse uniformity that could generate the reached results. Study of Ferreira *et al.* (2013) (26) investigated the profile particles by SEM of toothpastes and revealed that products of SiO2-based presenting higher roughness numbers. Yet, the presence of glycerin, cellulose and fluoride were able to prevent further mass loss by wearing structure of all studied toothpastes, that could explain, in parts, the statistical results (27). Composition of the resin matrix, matrix/particle interface, shape and size of the particles, degree of polymerization and hardness of the resin composites could interfere in the resistance to abrasion (28). According to Leinfelder *et al.* (29) the larger and more protruded the filler particles were, the more the energy generated by the processes of abrasion would be transmitted directly to the surrounding matrix, generating microcracks that could propagate and cause detachment of the particles, thereby increasing the roughness and potentiating the process of restoration wear even further. In this study, the resin composite Filtek Z350 was used, considered as a nanoparticulated resin, is compounded by BisGMA, UDMA, BisEMA and small quantities of TEGDMA (30). Their characteristics provide properties that are superior to those of the hybrid composites, such as better polishing, handling and capacity to maintain their anatomy for a longer period of time.

The interactions involving both factors (abrasive content type X nanocomposite resin) may provide other hypothesis regarding the reached outcomes. The incorporation of nanofillers improves the abrasive resistance, promoting a higher filler loading with consequent protection to softer matrix, which reduce the interparticle spacing and, at same time, enhancing the potential the material to abrasive effect (30). This fact could explain the images results, since the surface specimen, compounded by smaller nanosized particles are prone to break apart from each other more than the whole particle from matrix resin, making the surface with a less apparent “defect”, undetectable by the stereoscopic device supported by the enhanced optical properties of the tested composite, independent of the used abrasive. Thus, the lack of significant differences among the groups could be justified by all these inner properties, even after the simulated brushing challenge in the presence of different wearable compounds ( Table 1).

The ideal toothpaste would be one that promoted cleaning and polishing of tooth surfaces without occurrence of abrasion of the enamel and restorations. Nevertheless, it must be recognized that although toothpastes are very important due to the therapeutic functions attributed to them, they may also produce changes in the surface roughness of restorative materials and tooth enamel. Extending the time of evaluation, or even the quantity of cycles tested may perhaps, use of saliva, may result in the detection of more significant differences in the abrasiveness of these products on the tested resin. Therefore, further studies must be conducted with conventional and whitening toothpastes, using the most diverse qualitative and quantitative methods, simulating longer periods of use, to better elucidate the abrasive potential and its clinical manifestations.

Hence, although the toothpastes increased the surface roughness of the nanoparticle resin composite, no statistical differences among the whitening toothpastes tested was verified after the simulated brushing after15 days.
